# Intracranial residual lesions following early intensification in a patient with T-cell acute lymphoblastic leukemia: a case report

**DOI:** 10.1186/s12887-024-04790-3

**Published:** 2024-05-04

**Authors:** Yuichi Nagamatsu, Takeshi Isoda, Motoki Inaji, Jun Oyama, Daiki Niizato, Dan Tomomasa, Noriko Mitsuiki, Motoi Yamashita, Takahiro Kamiya, Kohsuke Imai, Hirokazu Kanegane, Tomohiro Morio, Masatoshi Takagi

**Affiliations:** 1 Department of Pediatrics and Developmental Biology, Tokyo Medical and Dental University, Tokyo, Japan; 2 Department of Neurosurgery, Tokyo Medical and Dental University, Tokyo, Japan; 3 Department of Diagnostic Radiology, Tokyo Medical and Dental University, Tokyo, Japan; 4 Department of Clinical Research Center, Tokyo Medical and Dental University, Tokyo, Japan; 5 Department of Community Pediatrics, Perinatal and Maternal Medicine, Tokyo Medical and Dental University, Tokyo, Japan; 6 Department of Pediatrics, National Defense Medical College, Tokorozawa, Japan; 7 Department of Child Health and Development, Tokyo Medical and Dental University, Tokyo, Japan; 8 Department of Pediatrics and Developmental Biology, Graduate School of Medical and Dental Sciences, Tokyo Medical and Dental University, 1-5-45 Yushima, Bunkyo-ku, Tokyo, 113-8519 Japan

**Keywords:** Case report, Central nervous system, ^11^C-methionine positron emission tomography, Induction failure, Minimal residual disease, T cell acute lymphoblastic leukemia

## Abstract

**Background:**

T-cell acute lymphoblastic leukemia (T-ALL) tends to involve central nervous system (CNS) infiltration at diagnosis. However, cases of residual CNS lesions detected at the end of induction and post early intensification have not been recorded in patients with T-ALL. Also, the ratio and prognosis of patients with residual intracranial lesions have not been defined.

**Case presentation:**

A 9-year-old boy with T-ALL had multiple intracranial tumors, which were still detected post early intensification. To investigate residual CNS lesions, we used ^11^C-methionine (MET)-positron emission tomography. Negative MET uptake in CNS lesions and excellent MRD status in bone marrow allowed continuing therapies without hematopoietic cell transplantation.

**Conclusions:**

In cases with residual lesions on imaging studies, treatment strategies should be considered by the systemic response, direct assessment of spinal fluid, along with further development of noninvasive imaging methods in CNS. Further retrospective or prospective studies are required to determine the prognosis and frequency of cases with residual intracranial lesions after induction therapy.

## Background

Leukemia is the most common cancer in children and the involvement of the central nervous system (CNS) increases the risk of CNS relapse [1]. Risk factors of CNS leukemia include infant onset, T-cell acute lymphoblastic leukemia (T-ALL), hyperleukocytosis, and chromosomal abnormalities including *KMT2A* rearrangements, *BCR-ABL1*, and *TCF3-PBX1* [1, 2]. These features as well as a traumatic tap at initial diagnosis also increase the risk of CNS relapse in ALL [1–3].

CNS status at initial diagnosis is classified as follows: CNS1, no leukemic cells in cerebrospinal fluid; CNS2, white blood cell (WBC) counts < 5/µL with leukemic cells; CNS3, WBC counts ≥ 5/µL with leukemic cells or neurological symptoms with imaging findings by computed tomography (CT) or magnetic resonance imaging (MRI) [4]. MRI is helpful to diagnose CNS leukemia in patients with neurological symptoms [5, 6]. The percentage of CNS3 leukemia with neurological symptoms and imaging findings in newly diagnosed ALL was estimated to be less than a few percent [1, 5].

Residual masses present in the CNS after induction or consolidation therapy are considered treatment failure (TF) [7]. TF affects later therapies, such as the decision to withdraw from protocol therapy and whether patients proceed to allogeneic hematopoietic cell transplantation (HCT) [7, 8]. The percentage of pediatric patients with induction failure (IF), defined by leukemic cells in bone marrow (BM) or extramedullary site after induction, was 2.4% (1041/44,017 cases) [8]. Among the 1041 cases, CNS status was available for 684 cases. Of these, CNS leukemia accounted for 6% (44/684) [8]. However, the ratio and prognosis of patients with isolated CNS residual masses by imaging have not been defined.

Here, we report a patient with T-ALL who developed multiple intracranial masses at diagnosis and had remaining residual lesions detected by follow-up MRI at the end of early intensification. We decided treatment strategy based on minimal residual disease (MRD) status in BM, assessment of spinal fluid, and whether ^11^C-methionine (MET) uptake in these CNS residual lesions.

## Case presentation

**Fig. 1:**
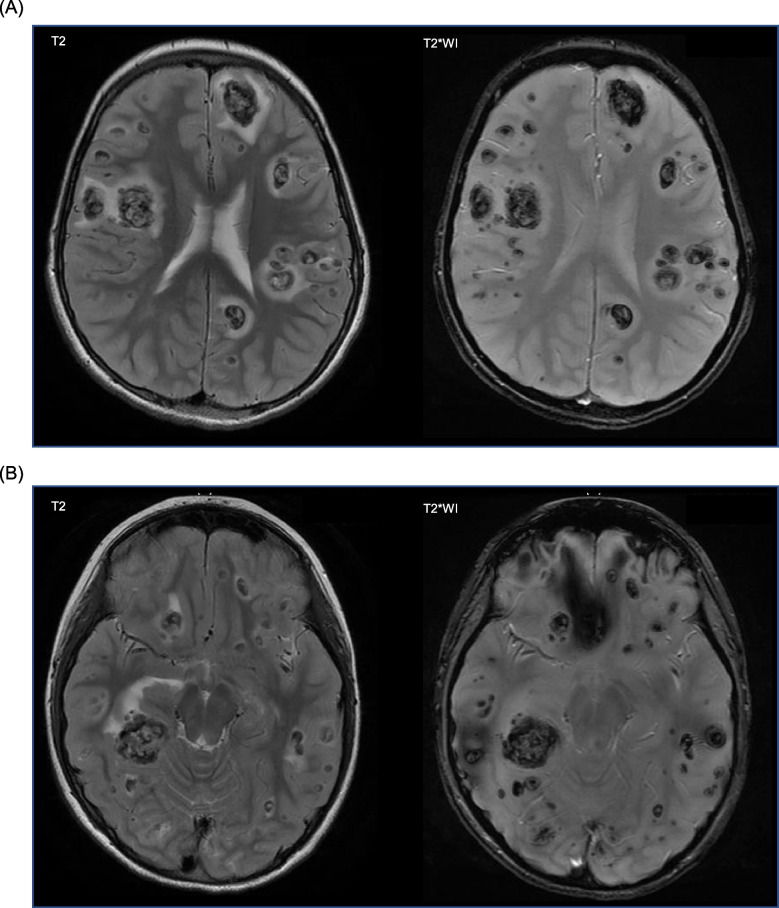
Multiple central nervous system infiltrations with hemorrhages on magnetic resonance images (MRI). **A** and **B** T2-weighted and T2*-weighted image (T2*WI) MRI shows heterogeneous signals with leukemic masses and hemorrhages. Edema is observed around multiple lesions

A 9-year-old boy was introduced to our hospital with somnolence, slurred speech, and petechiae. A complete blood count showed marked hyperleukocytosis (WBC 760 000/μL). Flow cytometric analysis showed positivity for CD1a, CD2, CD4, CD5, CD7, CD8, cytoplasmic CD3, and terminal deoxynucleotidyl transferase. G-banding revealed a normal karyotype and multiplex polymerase chain reaction (PCR) analysis did not detect fusion genes. Taken together, we diagnosed him with T-ALL. MRI revealed multiple intracranial masses with strong signal heterogeneity consisting of hemorrhage and leukemic infiltrations, which were consistent with definition of CNS3 (Fig. 1A and B).

**Fig. 2:**
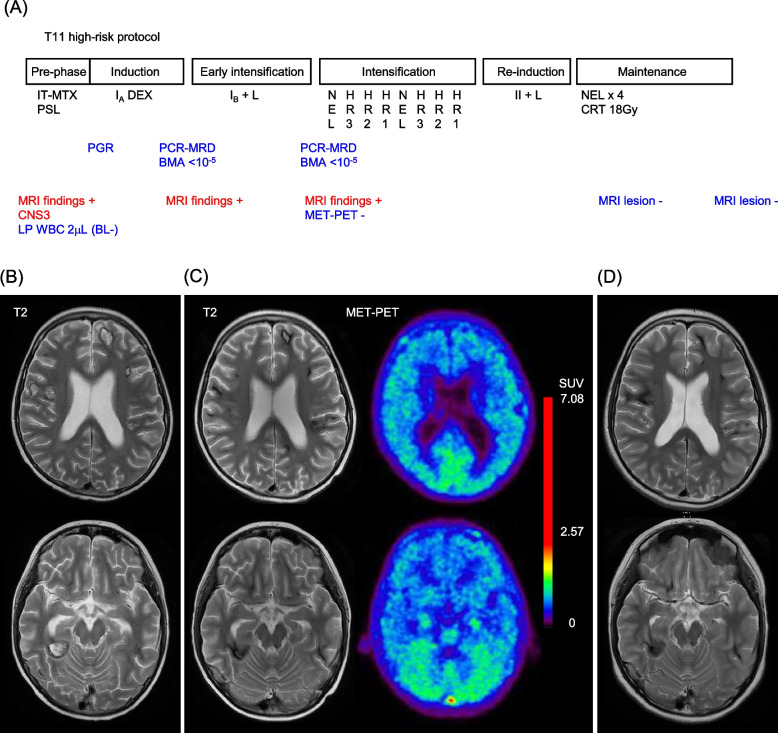
Treatment course, serial MRI, and 11C-methionine (MET) positron emission tomography (PET). **A** The Japan Leukemia/Lymphoma Study Group (JPLSG) T11 high-risk protocol with treatment response and CNS evaluation by imaging studies. **B** T2-weighted MRI showed residual multiple lesions at the end of induction. **C** At the end of early intensification, T2-weighted MRI shows two representative residual lesions at the same position as in (**B**). No significant MET uptake was detected. Scale bar indicates the standardized uptake value (SUV). **D** MRI images were captured two months after maintenance therapy. BL, blast; BMA, bone marrow analysis; CNS, central nervous system; CRT, cranial radiation therapy; HR, high risk; MET-PET, ^11^C-methionine positron emission tomography; MRD, minimal residual disease; NEL, nelarabine; IT, intrathecal; PSL, prednisolone

We started pre-phase therapy consisting of prednisolone (PSL) in accordance with the Japan Leukemia/Lymphoma Study Group T11 protocol (Fig. 2A) [9]. The number of lymphoblasts steadily decreased to 26 (< 1000)/μL in peripheral blood on day 8, and he was considered a PSL good responder (PGR) (Fig. 2A) [9]. There was no tumor lysis syndrome under the supportive therapy including hydration and rasburicase administration. His initial symptoms including somnolence and slurred speech gradually improved a week after starting corticosteroid. On day 8, we conducted initial intrathecal therapy (IT). Lumbar puncture (LP) showed a WBC count of 2/µL with mild cellular atypia by conventional cytospin. We completed the induction phase without intracranial hemorrhage. However, the patient had grade 3 hyponatremia due to cerebral salt wasting syndrome, and required additional sodium chloride supplementation by infusion for two weeks. On day 29, he also had grade 3 muscle weakness lower limb due to vincristine or corticosteroid myopathy and skipped vincristine once.

After day 18 of induction therapy, LP showed no evidence of malignant cells by cytospin. We did not perform flow cytometry analysis for spinal fluid in this case. The minimal residual disease (MRD) determined by PCR to monitor BM responses showed a reduction in lymphoblasts below 0.001% after induction therapy (Fig. 2A). However, MRI revealed multiple CNS masses (Fig. 2B). PCR-MRD for BM samples was maintained below 0.001%; however, MRI still showed multiple CNS lesions post early intensification (Fig. 2C).

To avoid invasive approaches, including needle biopsy or surgical resection, which might cause delaying further intensification therapies, we used ^11^C-methionine positron emission tomography (MET-PET) to investigate the activity of the residual lesions. MET-PET revealed no significant uptake, suggesting the low possibility of viable leukemic cells in residual regions (Fig. 2C). Thus, we classified him as belonging to a high-risk group, but not a very high-risk group requiring HCT, and he received continued chemotherapy including intensive IT, nelarabine, and cranial radiation therapy (CRT) in accordance with the T11 protocol [9]. The follow-up MRI revealed remission immediately after the maintenance therapy (Fig. 2D). At 18 months from the cessation of maintenance therapy and 46 months from onset, he has maintained a first remission and can attend regular school without neurological sequelae.

## Discussion and conclusions

We experienced a patient with T-ALL who showed multiple residual lesions in the CNS at the end of early intensification. PGR, good PCR-MRD status in BM, and no specific uptake of MET in the CNS allowed a continuation of chemotherapy with CRT, and the avoidance of HCT.

A meta-analysis of hematologic tumors, including pediatric to adult cases with intracranial sarcomas, reported 82 cases from 1999 to 2019 [10]. The report included 66 cases of AML, 10 cases of CML, 5 cases of APL, and 4 cases of ALL. Among them, three patients with ALL were relapsed cases [10]. Only two reports showed residual intracranial lesions after the start of treatment in primary ALL, both of which were Philadelphia chromosome positive ALL [11, 12]. The prognosis and appropriate treatment for cases of isolated residual CNS masses with excellent treatment responses in the non-CNS region have not been established because of the rarity of these cases.

The prognosis of IF in T-ALL was worse than that of B-ALL, with 10-year survival rates of 28 ± 3% and 41 ± 3%, respectively [8]. Historically, radiation therapy is beneficial for high-risk groups with T-ALL. The omission of prophylactic CRT in the T-ALL and CNS3 groups has not been established. Patients with CNS3 in the T-ALL group were ineligible in the recent AALL0434 study [13]. Taken together, chemotherapy with CRT was appropriate for our patient, even with negative MET uptake and good responses in the BM.

^18^F-fluorodeoxyglucose (FDG) is involved in glucose transport in tumors and noncancerous lesions including infections, inflammation, and the brain, especially the grey matter. Instead, MET has greater specificity for brain tumors using large amino acid transporter 2 [14]. MET-PET was developed to detect brain tumors including gliomas [14–16]. Compared with FDG, MET has an equivalent uptake in children with Hodgkin lymphoma and non-Hodgkin lymphoma at diagnosis and follow-up [17]. Interim PET has been feasible for evaluating T-lymphoblastic lymphoma [18]. Furthermore, MET-PET has been used to determine treatment responses in CNS lymphoma [19, 20]. Seo-Yeon et al. showed three response patterns correlated with prognosis, including low-, intermediate-, and high-risk, based on the interim tumor-to-normal tissue (T/N) ratio by MET uptake [19]. The interim T/N ratio in our patient was equivalent to the low-risk category, suggesting the low possibility of viable tumors in residual lesions.

However, there were limitations to this study. We could not perform MET-PET at onset due to his poor initial condition. If the lesion is small-scale or has low uptake of MET, it is difficult to distinguish the viability of residual tumor, which should be judged comprehensively in conjunction with other modalities. Thus, other clinical responses should be taken into account when decision-making. Finally, careful follow-up is required for our patient.

In conclusion, we experienced a patient with T-ALL and multiple residual lesions in the CNS at the end of early intensification. Further retrospective or prospective studies are required to determine the frequency of cases with intracranial residual lesions after induction therapy.

## Data Availability

The data that support the findings of this study are available on request from the corresponding author. The data are not publicly available due to privacy or ethical restrictions.

## Abbreviations

ALL: Acute lymphoblastic leukemia
AML: Acute myeloid leukemia
APL: Acute promyelocytic leukemia
BM: Bone marrow
CML: Chronic myelogenous leukemia
CNS: Central nervous system
CRT: Cranial radiation therapy
FDG: Fluorodeoxyglucose
HCT: Hematopoietic cell transplantation
IF: Induction failure
IT: Intrathecal
LP: Lumbar puncture
MET: Methionine
MRD: Minimal residual disease
MRI: Magnetic resonance imaging
PET: Positron emission tomography
PCR: Polymerase chain reaction
PGR: Prednisolone good responder
PSL: Prednisolone
TF: Treatment failure
WBC: White blood cells

## References

[1] Pui CH, Howard SC (2008). Current management and challenges of malignant disease in the CNS in paediatric leukaemia. Lancet Oncol.

[2] Pui CH, Campana D, Pei D, Bowman WP, Sandlund JT, Kaste SC (2009). Treating childhood acute lymphoblastic leukemia without cranial irradiation. N Engl J Med.

[3] Bürger B, Zimmermann M, Mann G, Kühl J, Löning L, Riehm H (2003). Diagnostic cerebrospinal fluid examination in children with acute lymphoblastic leukemia: significance of low leukocyte counts with blasts or traumatic lumbar puncture. J Clin Oncol.

[4] Mahmoud HH, Rivera GK, Hancock ML, Krance RA, Kun LE, Behm FG (1993). Low leukocyte counts with blast cells in cerebrospinal fluid of children with newly diagnosed acute lymphoblastic leukemia. N Engl J Med.

[5] Ranta S, Palomäki M, Levinsen M, Taskinen M, Abrahamsson J, Mellgren K (2017). Role of neuroimaging in children with acute lymphoblastic leukemia and central nervous system involvement at diagnosis. Pediatr Blood Cancer.

[6] Ulu EM, Töre HG, Bayrak A, Güngör D, Coşkun M (2009). MRI of central nervous system abnormalities in childhood leukemia. Diagn Interv Radiol.

[7] Buchmann S, Schrappe M, Baruchel A, Biondi A, Borowitz M, Campbell M (2022). Remission, treatment failure, and relapse in pediatric ALL: an international consensus of the Ponte-di-Legno Consortium. Blood.

[8] Schrappe M, Hunger SP, Pui CH, Saha V, Gaynon PS, Baruchel A (2012). Outcomes after induction failure in childhood acute lymphoblastic leukemia. N Engl J Med.

[9] Sato A, Hatta Y, Imai C, Oshima K, Okamoto Y, Deguchi T (2023). Nelarabine, intensive L-asparaginase, and protracted intrathecal therapy for newly diagnosed T-cell acute lymphoblastic leukaemia in children and young adults (ALL-T11): a nationwide, multicenter, phase 2 trial including randomisation in the very high-risk group. Lancet Haematol.

[10] Lee D, Omofoye OA, Nuño MA, Riestenberg RA, Shahlaie K (2021). Treatment outcomes of intracranial myeloid sarcomas: a meta-analysis. World Neurosurg.

[11] Cuvelier GD, Vitali AM, Ford JC, Dix DB (2008). Multiple intracranial tumors in Philadelphia chromosome positive acute lymphoblastic leukemia: successful treatment following aggressive supportive care, early cranial radiation, high dose chemotherapy and imatinib. Pediatr Blood Cancer.

[12] Hatakeyama N, Hori T, Yamamoto M, Inazawa N, Igarashi K, Tsutsumi H (2013). Multiple intracranial tumors in Philadelphia chromosome-positive acute lymphoblastic leukemia. Int J Hematol.

[13] Hayashi RJ, Winter SS, Dunsmore KP, Devidas M, Chen Z, Wood BL (2020). Successful outcomes of newly diagnosed T lymphoblastic lymphoma: results from Children’s Oncology Group AALL0434. J Clin Oncol.

[14] Glaudemans AWJM, Enting RH, Heesters MAAM, Dierckx RAJO, van Rheenen RWJ, Walenkamp AME (2013). Value of 11C-methionine PET in imaging brain tumours and metastases. Eur J Nucl Med Mol Imaging.

[15] Nariai T, Tanaka Y, Wakimoto H, Aoyagi M, Tamaki M, Ishiwata K (2005). Usefulness of L-[methyl-11C] methionine-positron emission tomography as a biological monitoring tool in the treatment of glioma. J Neurosurg.

[16] Nakano T, Tamura K, Tanaka Y, Inaji M, Hayashi S, Kobayashi D (2018). Usefulness of (11)C-methionine positron emission tomography for monitoring of treatment response and recurrence in a glioblastoma patient on bevacizumab therapy: a case report. Case Rep Oncol.

[17] Kaste SC, Snyder SE, Metzger ML, Sandlund JT, Howard SC, Krasin M (2017). Comparison of (11)C-methionine and (18)F-FDG PET/CT for staging and follow-up of pediatric lymphoma. J Nucl Medicine : official publication, Society of Nuclear Medicine.

[18] Gökbuget N, Wolf A, Stelljes M, Hüttmann A, Buss EC, Viardot A (2014). Favorable outcome in a large cohort of prospectively treated adult patients with T-lymphoblastic lymphoma (T-LBL) despite slowly evolving complete remission assessed by conventional radiography. Blood.

[19] Ahn SY, Kwon SY, Jung SH, Ahn JS, Yoo SW, Min JJ (2018). Prognostic significance of interim 11C-methionine PET/CT in primary central nervous system lymphoma. Clin Nucl Med.

[20] Miyakita Y, Ohno M, Takahashi M, Kurihara H, Katai H, Narita Y (2020). Usefulness of carbon-11-labeled methionine positron-emission tomography for assessing the treatment response of primary central nervous system lymphoma. Jpn J Clin Oncol.

